# Nutrition-Related Parameters and Imaging Severity of COVID-19 in Hospitalized Adults: A Secondary Analysis of a Prospective Multicenter Cohort

**DOI:** 10.3390/diagnostics16182917

**Published:** 2026-09-10

**Authors:** Andrzej Górecki, Anna Bronikowska, Zuzanna Szostak, Karolina Kołodziejczyk-Ryba, Ada Jankowska, Piotr Piech, Bartosz Borowski, Grzegorz Staśkiewicz

**Affiliations:** 1 Medical Diagnostic Center Voxel, Regional Hospital in Łańcut, 37-100 Łańcut, Poland; 2 Department of Correct, Clinical and Imaging Anatomy, Medical University of Lublin, 20-093 Lublin, Poland; 3 Chair of Traumatology, Orthopaedics and Rehabilitation, Medical University of Lublin, 20-093 Lublin, Poland; 4 Doctoral School of the Medical University of Lublin, 20-093 Lublin, Poland; 5 Department of Radiology, Medical University of Lublin, 20-093 Lublin, Poland

**Keywords:** COVID-19, nutrition-related parameters, dietary intake, albumin, total protein, bioimpedance analysis, disease severity, CT severity score, lung ultrasound, intensive care unit

## Abstract

**Background/Objectives:** Nutrition-related parameters may be associated with COVID-19 severity, but their relationships with complementary pulmonary imaging measures remain incompletely characterized. This study aimed to examine associations between selected nutrition-related parameters, pulmonary imaging severity, and clinical outcomes in hospitalized adults with COVID-19. **Methods:** This secondary exploratory analysis included 555 adults from a prospective multicenter cohort. Dietary intake assessed during the first week of hospitalization and derived from medical history, serum albumin and total protein concentrations, and selected bioimpedance parameters were examined in relation to computed tomography severity score (CTSS), lung ultrasonography (LUS) parameters, and selected clinical outcomes. **Results:** First-week hospital dietary intake was weakly inversely associated with CTSS (ρ = −0.110; *p* = 0.038), LUSSB (ρ = −0.274; *p* = 0.002), and LUSSBmax (ρ = −0.329; *p* < 0.001). History-derived dietary intake was associated with LUSSB (ρ = −0.253; *p* = 0.007) and LUSSBmax (ρ = −0.239; *p* = 0.011), but not with CTSS (ρ = −0.091; *p* = 0.120). Lower serum albumin was weakly associated with higher CTSS (ρ = −0.252; *p* = 0.008), whereas total protein was not (ρ = −0.199; *p* = 0.300). In unadjusted analyses, first-week hospital dietary intake, albumin, and total protein were associated with selected escalation-of-care outcomes, but not with in-hospital death. Nominal BIA–LUSSC associations were observed, but none remained statistically significant after correction for multiple testing. **Conclusions:** Selected nutrition-related parameters showed weak and non-uniform associations with pulmonary imaging severity in hospitalized adults with COVID-19. The findings do not establish independent prognostic relationships, and the exploratory bioimpedance results require confirmation in larger, appropriately designed studies.

## 1. Introduction

COVID-19, caused by SARS-CoV-2, was initially perceived primarily as a respiratory disease. However, it is now known that the infection may lead to multiorgan dysfunction involving, among others, the immune, cardiovascular, metabolic, and gastrointestinal systems. A severe course of the disease is associated with pronounced inflammation, metabolic disturbances, and the risk of multiorgan failure [1].

Patients hospitalized with COVID-19 may be vulnerable to deterioration of nutrition-related parameters during acute illness. According to the recommendations of the European Society for Clinical Nutrition and Metabolism (ESPEN), nutritional assessment and management should form part of supportive care in patients with SARS-CoV-2 infection [2].

Among laboratory nutrition-related parameters, serum albumin requires particularly cautious interpretation. Lower serum albumin concentrations have been associated with greater COVID-19 severity and poorer outcomes, but albumin is also influenced by the systemic inflammatory response and should not be interpreted as a specific measure of malnutrition [3].

Existing studies have often evaluated individual nutrition-related parameters. Their parallel assessment in relation to complementary pulmonary imaging measures may provide a broader characterization of how dietary intake, laboratory nutrition-related parameters, and body-composition measures co-occur with pulmonary disease severity.

Therefore, the aim of the present secondary analysis was to examine associations between selected nutrition-related parameters and COVID-19 severity in hospitalized adults, with particular emphasis on pulmonary involvement assessed by computed tomography and lung ultrasonography, as well as selected clinical outcomes.

## 2. Materials and Methods

### 2.1. Aim of the Study

The aim of the present study was to examine associations between selected nutrition-related parameters and COVID-19 severity, including pulmonary imaging findings, ICU admission, mechanical ventilation, and in-hospital death. The analysis was exploratory and was not designed to develop or validate a predictive model.

This study represents an extension of previous analyses conducted within the described project [4,5] and focuses on associations between selected nutrition-related parameters and COVID-19 severity. Earlier publications from the cohort focused primarily on CT- and lung-ultrasound-based assessment, whereas the present analysis addresses dietary intake, selected laboratory indicators, and bioimpedance parameters.

### 2.2. Study Design and Bioethical Approvals

The present study constitutes a secondary exploratory analysis conducted within a prospective, multicenter observational study performed in adults hospitalized due to SARS-CoV-2 acute infection. The parent study involved structured collection of clinical, laboratory, and imaging data during hospitalization.

The project was conducted from 27 October 2020 to 26 January 2022 in three participating centers: Łańcut, Jarosław, and Kościerzyna. Data were collected in a structured manner using a dedicated research system that enabled integration of clinical information, laboratory test results, and imaging data while ensuring patient anonymization.

The study was conducted in accordance with the applicable principles of scientific research and received a favorable opinion from the Bioethics Committee at the University of Rzeszów (Resolution No. 2/11/2020 of 6 November 2020). Written informed consent was obtained from all patients participating in the study.

### 2.3. Characteristics of the Participants

**Figure 1:**
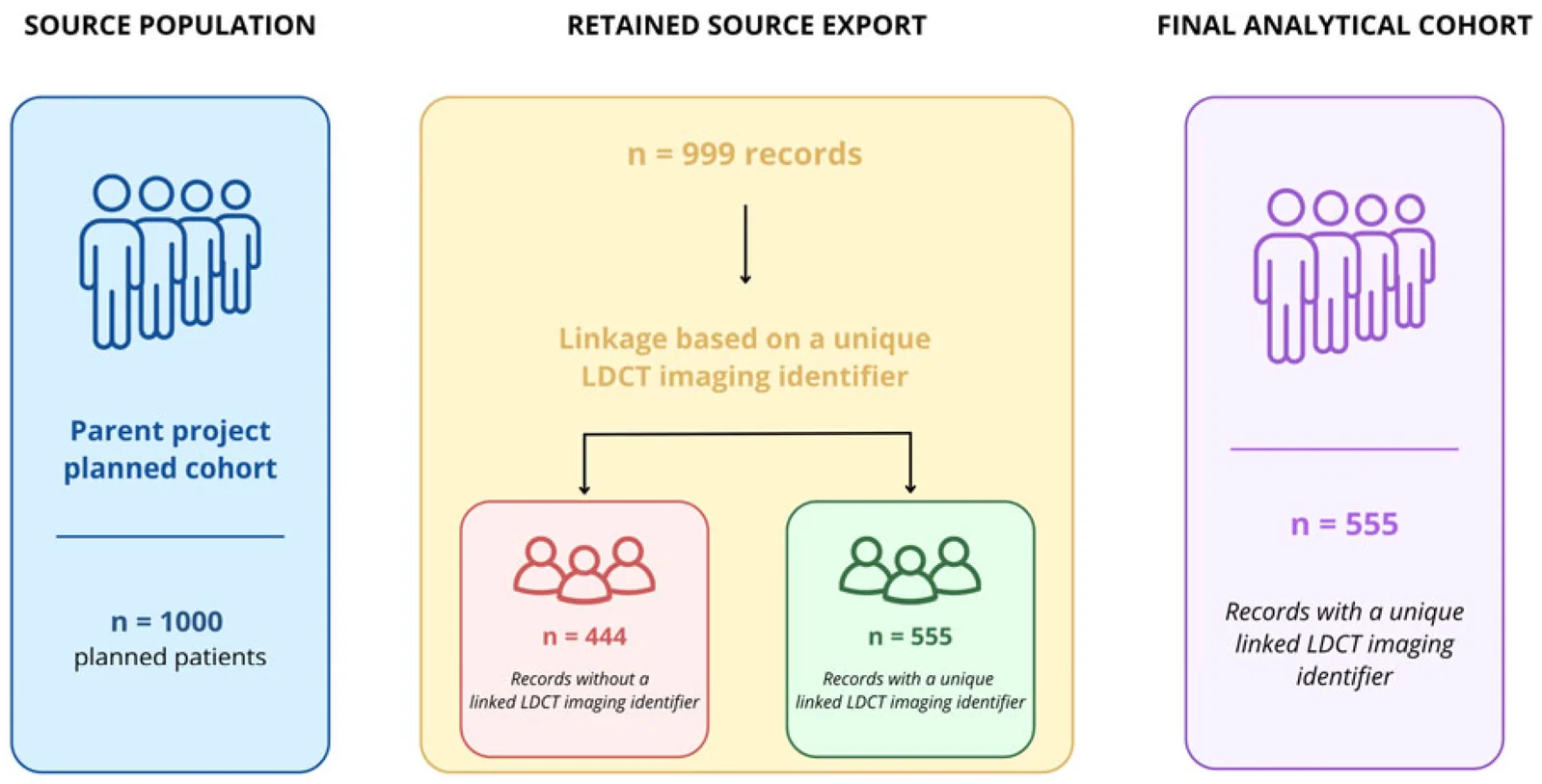
Flow diagram of the retained source records and linkage used to define the final analytical cohort.

The present secondary analysis included 555 adult patients hospitalized with COVID-19 for whom clinical data could be linked with the pulmonary imaging dataset using the available unique imaging identifier. The retained source dataset comprised 999 records, of which 555 had a unique linked NDTK imaging identifier and constituted the analytical cohort used in the present study. Data availability varied across individual variables and analyses. The process of final analytical cohort formation is presented in Figure 1.

The parent study enrolled adults hospitalized because of COVID-19. For the present secondary analysis, inclusion additionally required linkage of the retained clinical record with the pulmonary imaging dataset through the available unique NDTK identifier. The retained materials did not permit reliable reconstruction of specific clinical reasons for the absence of linkage in individual records.

The cohort analyzed in this study included patients with varying degrees of disease severity, ranging from mild to severe and critical cases. Demographic and clinical data, as well as information on the course of hospitalization, including the treatment received and the endpoints reached, were collected in a standardized manner within the research system.

### 2.4. Data Collection and Variables

Clinical, laboratory, and imaging data were collected in an organized, standardized manner, with use of a dedicated research system that enabled the integration and analysis of data from different centers. All data were anonymized prior to the analysis.

The scope of collected information included demographic and clinical data, as well as the results of additional tests obtained at patient’s hospital admission and during hospitalization. Information regarding the course of the disease, the treatment administered, and the endpoints was also included.

**Figure 2:**
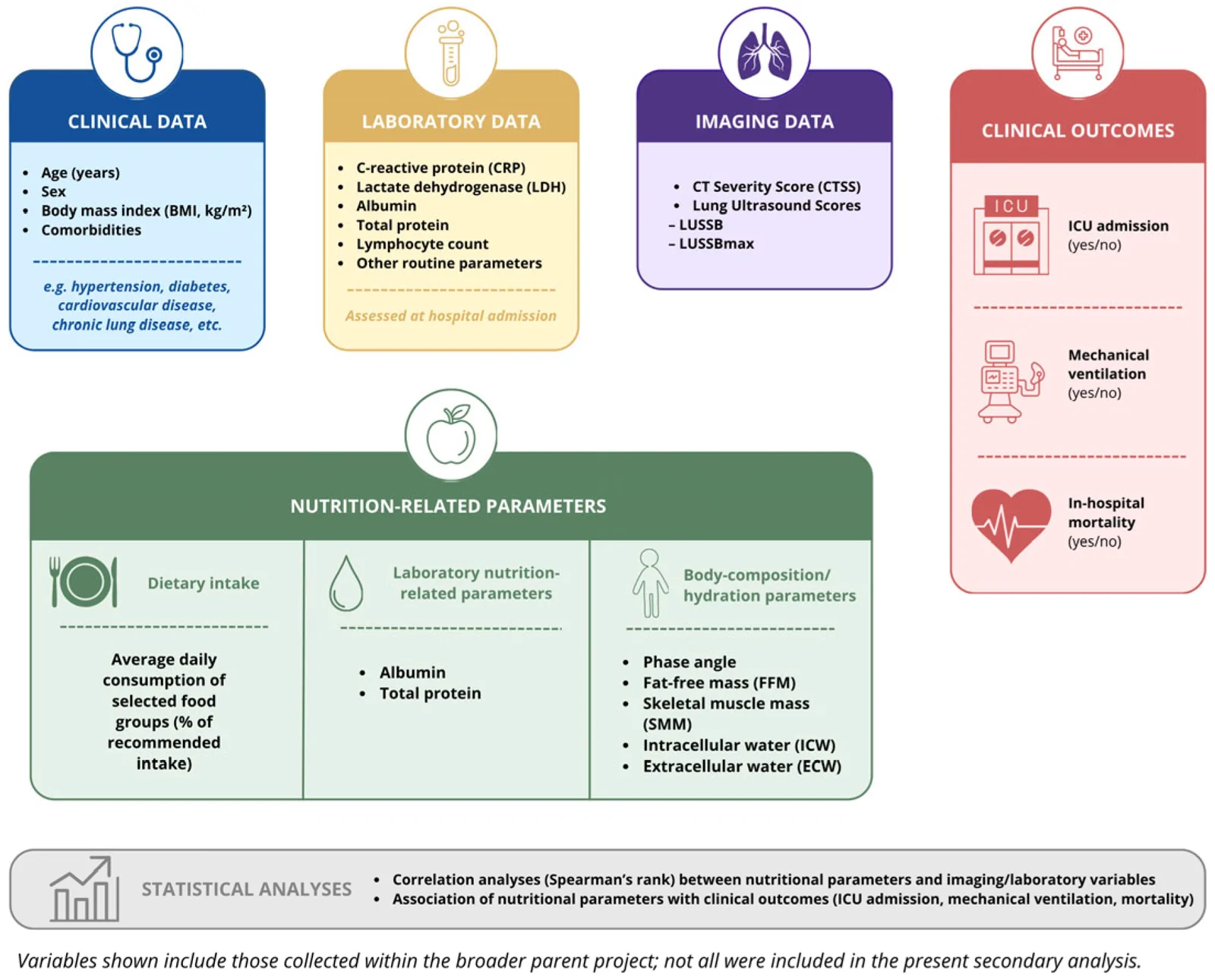
Overview of variables collected within the study project.

The clinical and laboratory variables collected within the study project, as well as selected parameters related to nutrition and COVID-19 severity, are presented schematically in Figure 2.

### 2.5. Assessment of Nutrition-Related Parameters

The analysis included selected nutrition-related parameters derived from dietary-intake assessment, laboratory measurements, and, in a subgroup of patients, bioimpedance analysis.

#### 2.5.1. Assessment of Dietary Intake

During hospitalization, dietary intake was assessed daily by the nursing staff caring for the patient on the ward. Intake was recorded semi-quantitatively by visual estimation of the proportion of the provided hospital food ration consumed, using predefined categories of 0%, 25%, 50%, 75%, and 100%. A value of 100% corresponded to the full hospital food ration offered to the patient and did not represent an individually calculated caloric or energy requirement. The same percentage scale was applied across the three participating centers. Two sources of information were analyzed: assessment during hospitalization and data obtained from the patients’ medical history. For the present analysis, first-week hospital dietary intake was calculated as the mean of the available daily assessments recorded during Days 1–7 of hospitalization. At least one assessment during this period was available for 391 patients; complete seven-day observations were available for 224 of these patients. No missing daily values were imputed. The percentage intake scale was an observational measure and was not equivalent to a validated nutritional-risk or malnutrition assessment tool.

#### 2.5.2. Nutrition-Related Laboratory Parameters

Selected laboratory parameters associated with nutritional and systemic clinical status were included in the analysis. The primary laboratory parameters evaluated in the present manuscript were serum albumin and total protein concentrations measured on Day 1 of hospitalization. These parameters were treated as nonspecific nutrition-related laboratory indicators rather than direct measures of malnutrition.

Day-1 serum albumin concentration was available for 119 patients and had a median value of 3.60 g/dL (IQR, 3.30–3.90), whereas Day-1 total protein was available for 30 patients, with a median of 6.75 g/dL (IQR, 6.30–6.90). Serum albumin was interpreted cautiously because its concentration may be influenced by systemic inflammation, hydration status, hepatic function, and the overall severity of acute illness.

#### 2.5.3. Bioimpedance Analysis

Bioimpedance measurements were available in the broader parent project for 137 patients; however, the BIA-imaging analyses that could be linked for the present secondary analysis comprised 38 patients. The analyzed parameters included extracellular water (ECW), intracellular water (ICW), phase angle (PA), standardized phase angle (SPA), and reactance (Xc). The retained historical BIA–LUSSC analysis additionally included a source variable labelled ‘Hydration’; its precise definition and unit could not be reliably reconstructed from the retained materials. The BIA component was considered exploratory because of the limited size of the linked analytical subgroup and the physiological interdependence of the analyzed parameters.

The analyzed parameters reflected dietary intake, systemic clinical status, hydration, and body composition.

**Figure 3:**
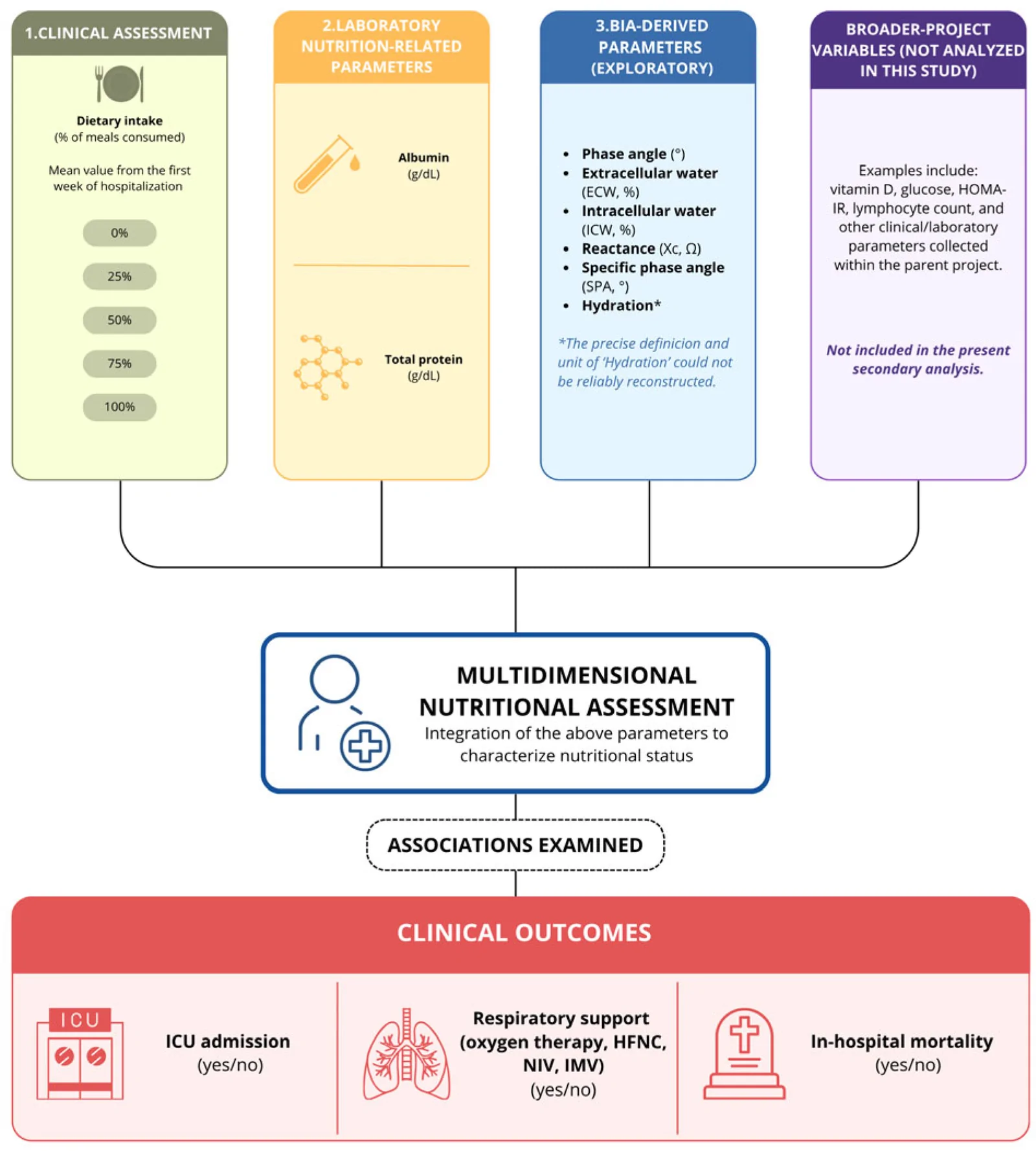
Overview of nutrition-related variables available within the broader study project. The present secondary analysis focused on dietary intake, serum albumin, total protein, and selected bioimpedance parameters.

Figure 3 presents the broader set of nutrition-related variables available within the study project. Not all variables shown were included in the present secondary analysis, which focused on dietary intake, serum albumin, total protein, and selected bioimpedance parameters.

### 2.6. Definition of Disease Severity

The level of disease severity was assessed on the basis of clinical and imaging parameters. The analysis included such factors as the need for hospitalization in the ICU, the use of mechanical ventilation, and in-hospital death as the main endpoints.

In addition, the extent of respiratory system involvement was assessed using imaging examinations, including computed tomography (CT) and lung ultrasonography.

For CT, the semiquantitative CT Severity Score (CTSS) was calculated as the sum of lobe-specific scores reflecting the extent of pulmonary parenchymal involvement. Each of the five lung lobes was scored from 0 to 5 according to the percentage of involvement, yielding a total score from 0 to 25; higher CTSS values indicated more extensive lung involvement.

Lung-ultrasound assessment included the Lung Ultrasound B-line Score (LUSSB), reflecting the burden of B-line artifacts, LUSSBmax, representing the maximum recorded B-line score, and the Lung Ultrasound Consolidation Score (LUSSC), reflecting pulmonary consolidations identified during lung ultrasonography. Higher values indicated more severe abnormalities. A first LUS examination was available for 110 patients and a second LUS examination for 109 patients; these overall availability counts should not be interpreted as analysis-specific denominators for individual dietary-intake-LUS correlations.

### 2.7. Statistical Analysis

Continuous variables were summarized using mean and standard deviation or median and interquartile range, as appropriate, whereas categorical variables were presented as counts and percentages. Associations between continuous variables were assessed using Pearson’s correlation coefficient or Spearman’s rank correlation coefficient, according to the analytical approach used for the respective variables. Between-group comparisons in the retained analyses were performed using Student’s *t*-test or the Mann-Whitney U test, as appropriate. The present reanalysis was based on available observations without imputation of missing values. For newly reconstructed complete-case analyses, only patients with available observations for both variables included in a given comparison were analyzed. Analysis-specific sample sizes are reported where they could be reliably determined; overall availability of an individual variable was not assumed to represent the denominator of a specific pairwise analysis. Statistical significance was assessed at an α level of 0.05. For the exploratory family of six BIA-LUSSC comparisons, a Bonferroni-adjusted significance threshold of α = 0.00833 (0.05/6) was additionally applied. The BIA analyses were considered exploratory because of the limited size of the linked analytical subgroup and the multiple physiologically related parameters examined.

During the manuscript creation, the Artificial Intelligence tool was used (Chat GPT, version GPT-5.6 Sol) in order to provide the correct translation of the text, as well as making the grammar as correct as it is possible.

## 3. Results

### 3.1. Characteristics of the Study Population

The final analytical cohort comprised 555 adults hospitalized with COVID-19, including 277 men, 275 women, and 3 individuals who did not declare a specific sex. The median age was 68 years (IQR, 58–75; range, 18–95 years). Data availability varied across individual variables and analyses.

CTSS was available for 512 patients, with a median value of 8 (IQR, 4–13). First-week hospital dietary intake was available for 391 patients and had a median value of 50.0% (IQR, 35.7–64.3). History-derived dietary intake was available for 319 patients, with a median of 50% (IQR, 50–75). Day-1 serum albumin concentration was available for 119 patients and had a median value of 3.60 g/dL (IQR, 3.30–3.90), whereas Day-1 total protein was available for 30 patients, with a median of 6.75 g/dL (IQR, 6.30–6.90). A first LUS examination was available for 110 patients; LUSSB had a median of 16.17 (IQR, 10.08–20.67) and LUSSC a median of 6.00 (IQR, 2.00–8.58). Admission characteristics, selected comorbidities and treatments, and the principal nutrition-related and imaging parameters are summarized in Table 1.

### 3.2. Relationship Between Dietary-Intake Measures

Both dietary-intake measures were available in 314 patients. First-week hospital dietary intake and history-derived dietary intake were moderately correlated (ρ = 0.411; *p* < 0.001). The median difference between hospital-observed and history-derived intake was 0 percentage points (IQR, −25 to +7.14). Hospital-observed intake was lower than the history-derived value in 132/314 patients (42.0%), identical in 85/314 (27.1%), and higher in 97/314 (30.9%). These findings indicate that the two measures were related but not interchangeable and were therefore analyzed separately.

### 3.3. Dietary Intake and Pulmonary Imaging Severity

In the complete-case CT analysis, first-week hospital dietary intake was weakly inversely correlated with CTSS (n = 358; ρ = −0.110; *p* = 0.038). First-week hospital dietary intake was also inversely correlated with LUSSB (ρ = −0.274; *p* = 0.002) and LUSSBmax (ρ = −0.329; *p* < 0.001).

History-derived dietary intake was not significantly associated with CTSS in the complete-case analysis (n = 292; ρ = −0.091; *p* = 0.120). However, inverse associations were observed with LUSSB (ρ = −0.253; *p* = 0.007) and LUSSBmax (ρ = −0.239; *p* = 0.011). All statistically significant correlations were weak, with the largest absolute coefficient observed between first-week hospital dietary intake and LUSSBmax (ρ = −0.329). The results are summarized in Table 2.

### 3.4. Laboratory Nutrition-Related Parameters and CTSS

In complete-case analyses, serum albumin concentration was weakly inversely correlated with CTSS (n = 111; ρ = −0.252; *p* = 0.008), indicating that lower albumin concentrations co-occurred with greater CT-defined pulmonary involvement. Total protein concentration was not significantly associated with CTSS (n = 29; ρ = −0.199; *p* = 0.300). The limited number of complete observations for the total-protein analysis should be considered when interpreting this result. The results are presented in Table 3.

### 3.5. Nutrition-Related Parameters and Clinical Outcomes

In the available unadjusted analyses, first-week hospital dietary intake was associated with ICU admission (*p* < 0.001), whereas history-derived dietary intake was not (*p* = 0.430). Neither first-week hospital dietary intake nor history-derived dietary intake was significantly associated with in-hospital death (*p* = 0.630 and *p* = 0.230, respectively).

Serum albumin concentration was associated with ICU admission and mechanical ventilation (*p* = 0.015 for both comparisons). Total protein concentration was also associated with ICU admission and mechanical ventilation (*p* = 0.009 for both comparisons). No significant associations with in-hospital death were identified for albumin or total protein. These unadjusted comparisons indicate statistical differences in selected nutrition-related parameters according to clinical outcome; however, the magnitude of these differences cannot be quantified from the retained analytical outputs. The results are summarized in Table 4.

Exact analysis-specific denominators, group-specific descriptive values, effect estimates, and 95% confidence intervals were not available from the retained endpoint-linked analytical outputs. The reported *p*-values derive from unadjusted comparisons and therefore indicate statistical evidence of between-group differences but do not quantify their magnitude. Missing observations were not imputed.

### 3.6. Exploratory Bioimpedance Analysis

The linked BIA-imaging analytical subgroup comprised 38 patients. No significant association was observed between BMI and CTSS (ρ = 0.237; *p* = 0.152).

In the original exploratory BIA–LUSSC analyses, nominal associations were observed for Hydration (ρ = 0.376; *p* = 0.029), ECW% (r = 0.414; *p* = 0.015), ICW% (r = −0.414; *p* = 0.015), PA (r = −0.353; *p* = 0.041), Xc (ρ = −0.403; *p* = 0.019), and SPA (r = −0.397; *p* = 0.020). Because these six comparisons represented a family of physiologically related exploratory tests, a Bonferroni-adjusted significance threshold of α = 0.00833 was applied. None of the six associations remained statistically significant after correction for multiple testing. Accordingly, the BIA findings were considered exploratory and hypothesis-generating. The results are depicted in Table 5.

**Figure 4:**
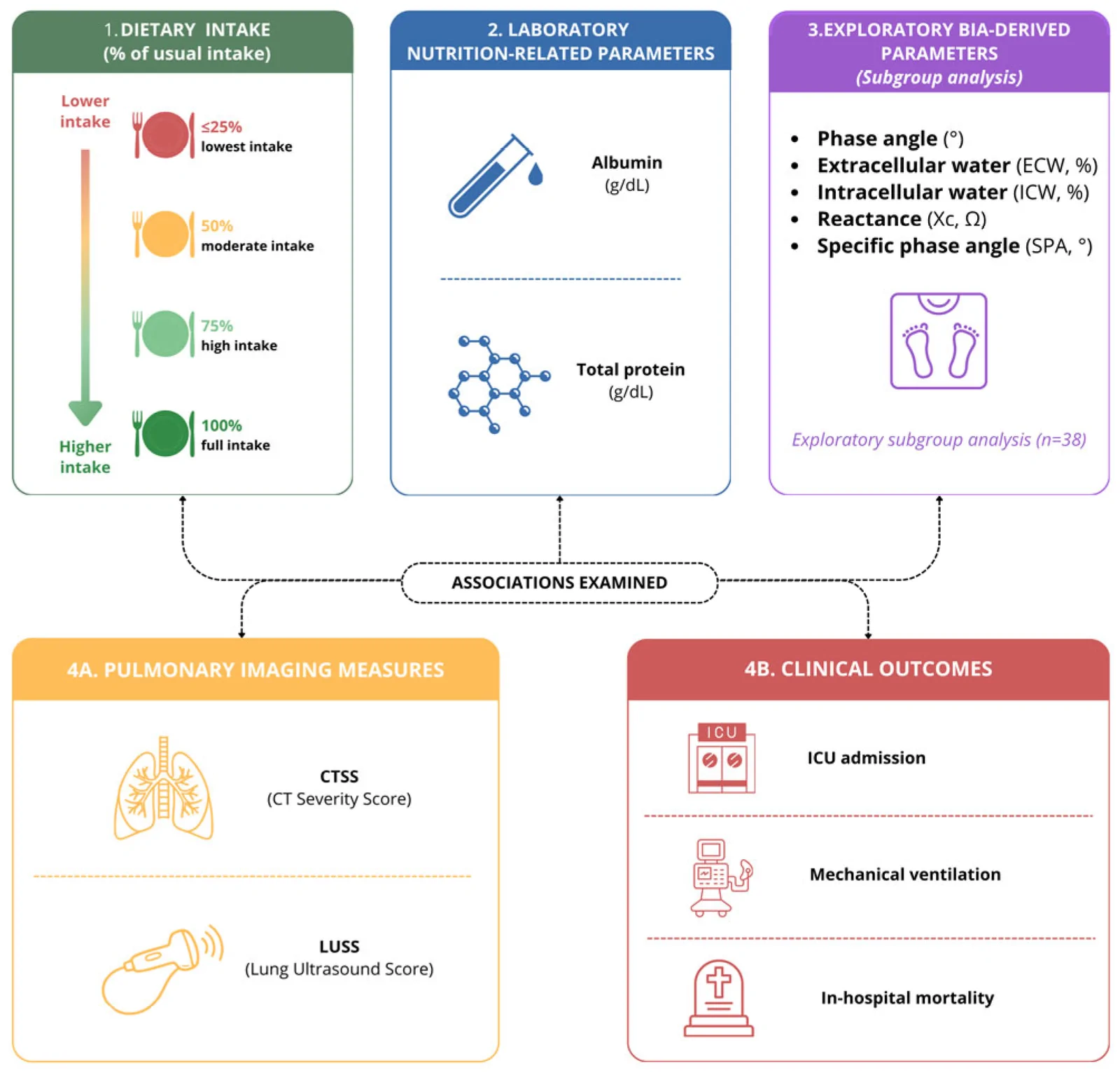
Schematic summary of the principal associations evaluated between nutrition-related parameters, pulmonary imaging severity, and clinical outcomes in the present secondary analysis. Relationships are presented as associations and should not be interpreted as causal. Bioimpedance-derived findings were exploratory and did not remain statistically significant after correction for multiple testing.

The principal associations evaluated in the present secondary analysis are summarized schematically in Figure 4.

## 4. Discussion

In this secondary analysis of a prospective multicenter cohort of adults hospitalized with COVID-19, selected nutrition-related parameters showed weak and non-uniform associations with pulmonary imaging severity and selected clinical outcomes. First-week hospital dietary intake was weakly associated with CTSS and with LUS-derived measures, whereas history-derived dietary intake was associated with LUS parameters but not with CTSS. Lower serum albumin, but not total protein, was associated with greater CT-defined pulmonary involvement. In the available unadjusted analyses, first-week hospital dietary intake, albumin, and total protein were associated with selected escalation-of-care outcomes, whereas no significant associations with in-hospital mortality were identified. Exploratory associations between bioimpedance parameters and LUSSC did not remain statistically significant after correction for multiple testing. Overall, the findings indicate that the observed relationships were modest and should be interpreted as associations rather than evidence of independent prognostic effects.

### 4.1. Dietary Intake and Pulmonary Imaging Severity

The present study identified weak and non-uniform associations between dietary intake and pulmonary imaging severity. First-week hospital dietary intake was weakly inversely associated with CTSS and with both analyzed LUS parameters, whereas history-derived dietary intake was associated with LUSSB and LUSSBmax but not with CTSS in the complete-case analysis. The largest observed correlation was between first-week hospital dietary intake and LUSSBmax (ρ = −0.329), indicating that the magnitude of the imaging associations was limited.

The two dietary-intake measures were moderately correlated but were not interchangeable. Their differing associations with CT and LUS may therefore reflect, at least in part, differences in the clinical periods and aspects of food intake captured by each assessment. Importantly, dietary intake assessed during hospitalization may itself have been affected by acute disease severity, and the present observational analysis cannot establish whether reduced intake preceded or resulted from clinical deterioration.

Previous studies have reported that malnutrition risk, adverse nutrition-related parameters, and nutrition-related complaints are common in hospitalized patients with COVID-19 and may accompany greater morbidity or poorer clinical outcomes [6,7,8].

Hospital nutritional management has also been investigated in relation to respiratory support and hospitalization outcomes [9]. However, the weak magnitude and inconsistency of the associations observed across imaging modalities in the present study argue against interpreting dietary intake as an independent marker of pulmonary disease severity.

### 4.2. Serum Albumin and Total Protein in Relation to Pulmonary Imaging Severity and Clinical Outcomes

The interpretation of laboratory nutrition-related parameters in acute COVID-19 requires particular caution. Serum albumin is a nonspecific marker influenced by inflammation, hydration status, liver function, and nutrition-related factors, and should therefore not be regarded as a direct measure of malnutrition [3,10].

Previous pooled evidence has associated lower serum albumin concentrations with greater COVID-19 severity and poorer outcomes [3]. Nevertheless, because albumin is strongly influenced by the acute inflammatory response, the observed association cannot be attributed specifically to nutritional status. In contrast, total protein was not significantly associated with CTSS in the complete-case analysis. This analysis was based on a substantially smaller number of complete observations and should therefore be interpreted cautiously.

Nutritional considerations in frail patients with COVID-19 further underscore the close interaction between nutrition-related abnormalities and the overall clinical condition [11]. In particular, the absence of multivariable adjustment for inflammatory burden, comorbidities, treatment, and overall disease severity prevents determination of whether the observed associations were independent of the broader clinical condition. Nevertheless, these findings identify potentially relevant relationships between nutrition-related parameters and clinical outcomes that merit confirmation in prospective studies specifically designed to assess their independent prognostic value.

### 4.3. Complementary Imaging Assessment and Exploratory Bioimpedance Findings

An important feature of the present analysis is the assessment of nutrition-related parameters in relation to two complementary approaches to pulmonary imaging. CTSS and LUS-derived scores have both been investigated as quantitative measures of pulmonary disease severity and clinical outcome in COVID-19 [12,13,14]. In the present study, the non-uniform relationships observed across CT- and LUS-derived parameters suggest that the two imaging approaches may capture partly different aspects of pulmonary involvement.

The exploratory bioimpedance analysis provided additional information on body-fluid distribution and cellular characteristics. Several nominal associations with LUSSC were observed for parameters related to extracellular and intracellular water and cellular integrity. Such parameters have previously been interpreted in relation to fluid distribution and cellular condition [15,16]. However, the analyzed BIA parameters are physiologically interrelated, and the analysis was performed in a small linked subgroup.

After correction for the six BIA–LUSSC comparisons, none of the nominal associations remained statistically significant. Accordingly, these findings should not be interpreted as independent or mutually reinforcing evidence of an association between bioimpedance characteristics and pulmonary severity. They should instead be regarded as hypothesis-generating observations requiring confirmation in larger, appropriately designed studies.

### 4.4. Potential Clinical Relevance and Interpretation

The practical relevance of the observed associations should be interpreted in light of their generally weak magnitude. Dietary intake and selected laboratory nutrition-related parameters co-occurred with pulmonary imaging severity or selected escalation-of-care outcomes, but the present analyses do not demonstrate that these variables provide independent prognostic information or improve individual risk stratification. Similar observations have been reported in studies linking nutritional risk and difficulties with food intake to poorer outcomes in patients with COVID-19 [7,8].

ESPEN guidance emphasizes nutritional assessment and management as part of supportive care in patients with SARS-CoV-2 infection [2]. The present findings should not, however, be interpreted as evidence that the analyzed parameters independently predict subsequent clinical deterioration.

Nutritional problems may also persist during recovery after severe COVID-19 and intensive care [17]. In this context, the present results support consideration of nutrition-related parameters as part of the broader clinical characterization of hospitalized patients rather than as stand-alone prognostic markers.

### 4.5. Pathophysiological and Clinical Context

The observed associations should also be considered within the broader interaction between inflammation, metabolism, and nutrition in COVID-19. Previous reports have described bidirectional relationships in which malnutrition and metabolic disturbances may coexist with and be influenced by the inflammatory response and clinical severity [18,19]. In this context, the nutrition-related parameters evaluated in the present study may reflect components of the broader systemic response to acute disease rather than isolated nutritional effects.

Inflammatory markers have also been associated with frailty and in-hospital mortality in older patients with COVID-19 [20]. These considerations reinforce the need to interpret the observed nutrition-related associations within the overall clinical condition rather than as independent nutritional or prognostic effects.

### 4.6. Strengths, Temporal Context, and Limitations

An important strength of the present study is its basis in a prospectively assembled multicenter cohort integrating clinical, laboratory, dietary-intake, and pulmonary imaging information. The parallel consideration of CT and LUS findings together with several nutrition-related domains provides a multidimensional perspective that is not captured by analyses focused on a single laboratory or nutritional parameter. Several limitations should nevertheless be considered. First, the present study was a secondary exploratory analysis, and the reported clinical-outcome analyses were unadjusted. Residual confounding by comorbidities, inflammatory burden, treatment, and overall disease severity therefore cannot be excluded.

The temporal relationship between dietary intake and disease progression cannot be established. Dietary intake during hospitalization represented the mean of available observations during the first week, and reduced intake may have resulted from clinical deterioration rather than preceded it. The secondary analysis did not use a prespecified hierarchy of hypotheses, and no dedicated power calculation was available for the present secondary analyses.

The percentage-based dietary-intake measures and the analyzed laboratory parameters do not constitute validated measures of malnutrition. Serum albumin is additionally influenced by acute inflammation, hydration status, and other clinical factors. Standardized nutritional-risk or malnutrition instruments such as NRS-2002, MUST, MNA-SF, or GLIM criteria were not available.

Data availability varied across variables and analyses. Available observations were analyzed without imputation, resulting in analysis-specific sample sizes. Exact denominators could be reconstructed for the principal complete-case CTSS analyses, whereas they could not be reliably determined for some retained LUS and clinical-outcome comparisons. The BIA–imaging analysis was restricted to 38 participants with available linked measurements; therefore, this subgroup may not be representative of the overall cohort, and selection bias cannot be excluded. Factors influencing fluid distribution may also have affected these measurements. None of the nominal BIA-LUSSC associations remained statistically significant after correction for multiple testing. These findings should consequently be regarded as exploratory and require confirmation in larger cohorts with prospectively integrated BIA and imaging assessments.

Center-specific distribution of the final linked analytical cohort could not be reliably reconstructed from the retained dataset, and center effects were therefore not evaluated in the present secondary analysis. Recruitment occurred between October 2020 and January 2022 and therefore encompassed different phases of the COVID-19 pandemic. Patient-level information on SARS-CoV-2 variants and vaccination status was not available for the present secondary analysis, and temporal changes in treatment practice could not be comprehensively accounted for. The findings should therefore be interpreted in the context of hospitalized COVID-19 during the study period rather than directly extrapolated to contemporary variants, vaccination patterns, or treatment pathways.

## 5. Conclusions

In hospitalized adults with COVID-19, selected nutrition-related parameters showed weak and non-uniform associations with pulmonary imaging severity. Lower first-week hospital dietary intake and lower serum albumin concentration were weakly associated with greater CT-defined pulmonary involvement, whereas history-derived dietary intake and total protein were not significantly associated with CTSS in complete-case analyses. Dietary-intake measures were also associated with selected LUS parameters. In the available unadjusted analyses, first-week hospital dietary intake, serum albumin, and total protein were associated with selected escalation-of-care outcomes, whereas no significant associations with in-hospital mortality were identified. These findings do not establish independent prognostic effects.

The exploratory bioimpedance findings did not remain statistically significant after correction for multiple testing and should therefore be regarded as hypothesis-generating. Overall, the results support further investigation of nutrition-related parameters alongside complementary quantitative pulmonary imaging approaches using standardized nutritional assessment and appropriately designed multivariable analyses.

## Figures and Tables

**Table 1:** Baseline characteristics and availability of principal study variables.

Domain	Variable	Value
Cohort	Total analytical cohort	555
Demographics	Sex	Male 277 (49.9%); Female 275 (49.5%); Other 3 (0.5%)
Demographics	Age, years	Median 68 (IQR 58–75); range 18–95
Admission	SpO_2_	n = 395; median 94 (IQR 90–96)
Admission	Temperature, °C	n = 396; median 36.7 (IQR 36.5–37.0)
Admission	Blood pressure, mmHg	n = 356; SBP 130 (117.75–140); DBP 80 (70–88)
Comorbidities *	Hypertension	153/311 (49.2%)
Comorbidities *	Diabetes	71/311 (22.8%)
Comorbidities *	Coronary artery disease/MI/PCI/CABG	37/311 (11.9%)
Comorbidities *	Heart failure	24/311 (7.7%)
Comorbidities *	Asthma/COPD	18/311 (5.8%)/9/311 (2.9%)
Relevant treatments †	Antibiotics/remdesivir/ systemic corticosteroids	243/396 (61.4%)/232/396 (58.6%)/225/396 (56.8%)
Imaging	CTSS	n = 512; median 8 (IQR 4–13)
Dietary intake	First-week hospital dietary intake	n = 391; median 50.0% (IQR 35.7–64.3)
Dietary intake	History-derived dietary intake	n = 319; median 50% (IQR 50–75)
Laboratory	Albumin, Day 1, g/dL	n = 119; median 3.60 (IQR 3.30–3.90)
Laboratory	Total protein, Day 1, g/dL	n = 30; median 6.75 (IQR 6.30–6.90)

* *Comorbidity percentages use the* 311 *patients with available comorbidity history as the denominator. † Treatment percentages use the* 396 *patients with available treatment data as the denominator. Data availability varied by variable. Length of stay is not presented because patient-level length-of-stay data for the final cohort of* 555 *could not be reliably reconstructed.*

**Table 2:** Associations between dietary-intake measures and pulmonary imaging severity.

Dietary-Intake Measure	Imaging Parameter	Analysis-Specific n	Correlation	*p*-Value
First-week hospital dietary intake	CTSS	358	ρ = −0.110	0.038
First-week hospital dietary intake	LUSSB	NR	ρ = −0.274	0.002
First-week hospital dietary intake	LUSSBmax	NR	ρ = −0.329	<0.001
History-derived dietary intake	CTSS	292	ρ = −0.091	0.120
History-derived dietary intake	LUSSB	NR	ρ = −0.253	0.007
History-derived dietary intake	LUSSBmax	NR	ρ = −0.239	0.011

*NR, not reliably reconstructable from the retained linked datasets. Overall first-LUS availability was n = 110; this value must not be presented as the denominator of a specific dietary-intake × LUS correlation. Complete-case n values are reported for the reconstructed CTSS analyses.* ρ—Spearman rank correlation coefficient; CTSS—computed tomography severity score; LUSSB—lung ultrasound B-line score; LUSSBmax—maximum lung ultrasound B-line score. Complete-case n values are reported for reconstructed CTSS analyses. Overall first-LUS availability was n = 110 and must not be interpreted as the denominator of a specific dietary-intake–LUS correlation; exact analysis-specific LUS denominators could not be reliably reconstructed from the retained linked datasets.

**Table 3:** Associations between laboratory nutrition-related parameters and CTSS.

Variable	Analysis-Specific n	Correlation with CTSS	*p*-Value
Albumin, Day 1 (g/dL)	111	ρ = −0.252	0.008
Total protein, Day 1 (g/dL)	29	ρ = −0.199	0.300

ρ—Spearman rank correlation coefficient; CTSS—computed tomography severity score. Complete-case analyses; no missing observations were imputed.

**Table 4:** Unadjusted associations between nutrition-related parameters and selected clinical outcomes.

Parameter	Clinical Outcome	*p*-Value
First-week hospital dietary intake	ICU admission	<0.001
First-week hospital dietary intake	In-hospital death	0.630
History-derived dietary intake	ICU admission	0.430
History-derived dietary intake	In-hospital death	0.230
Albumin, Day 1	ICU admission	0.015
Albumin, Day 1	Mechanical ventilation	0.015
Total protein, Day 1	ICU admission	0.009
Total protein, Day 1	Mechanical ventilation	0.009

**Table 5:** Exploratory associations involving bioimpedance-derived parameters in the linked BIA–imaging subgroup (n = 38).

BIA Parameter	Correlation with LUSSC	Nominal *p*-Value
Hydration	ρ = 0.376	0.029
ECW (%)	r = 0.414	0.015
ICW (%)	r = −0.414	0.015
PA	r = −0.353	0.041
Xc	ρ = −0.403	0.019
SPA	r = −0.397	0.020

## Data Availability

The patient-level dataset is not publicly available because it originated from a multicenter clinical project and is subject to institutional and ethical restrictions. The present secondary analysis was based on the study dataset and the available statistical summaries.
